# An international survey on hypoglycemia among insulin-treated type I and type II diabetes patients: Turkey cohort of the non-interventional IO HAT study

**DOI:** 10.1186/s12902-018-0238-2

**Published:** 2018-02-13

**Authors:** Rıfat Emral, Tamer Tetiker, Ibrahim Sahin, Ramazan Sari, Ahmet Kaya, İlhan Yetkin, Sefika Uslu Cil, Neslihan Başcıl Tütüncü, on behalf of the IO HAT investigator group

**Affiliations:** 1 Department of Endocrinology and Metabolic Diseases, Ankara University, Faculty of Medicine, İbn-i Sina Hospital, Academic Region M1/09, Samanpazarı, 06100 Ankara, Turkey; 2 Faculty of Medicine, Department of Endocrinology and Metabolic Diseases, Çukurova University, Adana, Turkey; 3 Endocrinology and Metabolism Department, Inonu University School of Medicine, Malatya, Turkey; 4 Division of Endocrinology and Metabolism, School of Medicine, Akdeniz University, Antalya, Turkey; 5 Division of Endocrinology and Metabolism, Meram School of Medicine, Necmettin Erbakan University, Konya, Turkey; 6 Division of Endocrinology and Metabolism, School of Medicine, Gazi University, Ankara, Turkey; 7 Medical Department, Novo Nordisk, Istanbul, Turkey; 8 Division of Endocrinology and Metabolism, School of Medicine, Baskent University, Ankara, Turkey

**Keywords:** Diabetes, Turkey, Hypoglycemia, IO HAT, Insulin, Non-interventional

## Abstract

**Background:**

Limited real-world data are currently available on hypoglycemia in diabetes patients. The International Operations Hypoglycemia Assessment Tool (IO HAT) study was designed to estimate hypoglycemia in insulin-treated type I (T1DM) and type II (T2DM) diabetes mellitus patients from 9 countries. The data from Turkey cohort are presented here.

**Methods:**

A non-interventional study to determine the hypoglycemia incidence, retrospectively and prospectively, in Turkish T1DM and T2DM patients using a 2-part self-assessment questionnaire.

**Results:**

Overall, 2348 patients were enrolled in the Turkey cohort (T1DM = 306 patients, T2DM = 2042 patients). In T1DM patients, 96.8% patients reported hypoglycemic events (Incidence rate [IR]: 68.6 events per patient-year [ppy]), prospectively, while 74.0% patients reported hypoglycemic events (IR: 51.7 events ppy), retrospectively. In T2DM patients, 95.9% patients (IR: 28.3 events ppy) reported hypoglycemic events, prospectively, while 53.6% patients (IR: 23.0 events ppy) reported hypoglycemic events, retrospectively. Nearly all patients reported hypoglycemia during the prospective period.

**Conclusions:**

This is a first patient-reported dataset on hypoglycemia in Turkish, insulin-treated diabetes patients. A high incidence of patient-reported hypoglycemia confirms that hypoglycemia remains under-estimated. Hypoglycemia increased healthcare utilization impacting patients’ quality of life. Hypoglycemia remains a common side effect with insulin-treatment and strategies to optimize therapy and reduce hypoglycemia occurrence in diabetes patients are required.

**Trial registration:**

Clinicaltrials.gov**,** NCT02306681 (Date of registration: 12 Nov 2014; retrospectively registered).

**Electronic supplementary material:**

The online version of this article (10.1186/s12902-018-0238-2) contains supplementary material, which is available to authorized users.

## Background

Insulin therapy remains integral to treatment of type I diabetes mellitus (T1DM) and long-term type II diabetes mellitus (T2DM) [1]. A good glycemic control is essential to minimize development of microvascular complications and macrovascular events [2]. Hypoglycemia is the main hurdle for achieving optimal glycemic control in patients on insulin therapy [3]. Development of strategies or therapies to control hypoglycemia is important to help individuals achieve glycemic targets [4]. Achieving optimum glycemic control following the diagnosis of T2DM is vital to improving clinical outcomes, yet many patients and clinicians are hesitant to initiate and intensify insulin therapy. Reasons for this are manifold including lack of time, clinical expertise and patient understanding. However, considerable progress can be achieved with patient education and awareness programs soon after diagnosis [5].

Despite the apparent high risk of hypoglycemia, only a few studies have been conducted to evaluate the incidence rate of hypoglycemia in a real-world setting. Hypoglycemia is commonly reported in a clinical trial context; however, these studies seldom reflect real-life clinical practice due to rigorous inclusion and exclusion criteria involved, and continuous treatment and follow-up.

Increasing evidence on growing incidence of diabetes in low- and middle-income countries has been reported [6]. According to two population-based studies, the prevalence of T2DM in Turkey increased from 7.2% to 16.5% within 12 years [7, 8]. Current knowledge on hypoglycemia comes from a few studies in North American or European populations and very limited data are available on hypoglycemia in Turkish diabetes patients [9, 10].

This paper describes the results from the Turkey cohort of the International Operations Hypoglycemia Assessment Tool (IO HAT) study which was conducted in 9 countries. The IO HAT study builds on findings from the global HAT study that was conducted in 24 countries [11]. The IO HAT study is an observational study aimed at enhancing the clinical understanding of hypoglycemia, and its clinical, social and economic consequences. In turn, this will help to identify cost-effective solutions to improve blood glucose control and Quality of Life (QOL) for patients with diabetes. The current study aims to assess hypoglycemia retrospectively and prospectively among insulin treated patients with T1DM or T2DM.

## Methods

### Study design

The Turkey cohort of the IO HAT study was a non-interventional, multi-center, 6-month and 4-week retrospective and 4-week prospective study to assess hypoglycemia in insulin-treated diabetes patients.

**Fig. 1:**
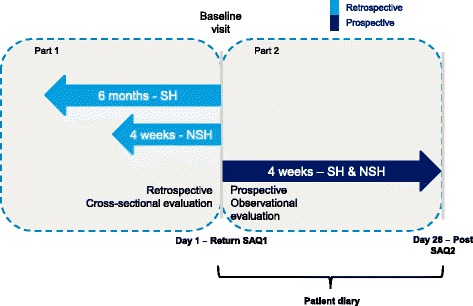
IO HAT Study design. Severe hypoglycemia: an event requiring assistance of another person to actively administer carbohydrate, glucagon, or other resuscitative actions; Non-severe hypoglycemia: documented symptomatic (symptoms and blood glucose measurement ≤3.9 mmol/L [70 mg/dL]) and probable symptomatic (symptoms only). NSH = non-severe hypoglycemia; SH = severe hypoglycemia; SAQ = self-assessment questionnaire

The study was carried out at 92 sites in Turkey. The study design is described in Fig. 1. The study protocol and assessments were conducted in accordance with the Declaration of Helsinki (2013) and the Guidelines for Good Pharmacoepidemiology Practices (2007), and approved by an Ethics Committee. All study materials were translated into Turkish, and data obtained were translated back into English for analysis.

### Study population

The study was conducted in male or female T1DM or T2DM patients treated with insulin for more than 12 months and who were 18 years or older, ambulatory, literate, and had given informed consent to participate in the study. To minimize selection bias, eligible patients were enrolled consecutively during routine clinic visits.

### Assessments

This study comprised of a two-part self-assessment questionnaire (SAQ) including a retrospective cross-sectional evaluation (SAQ1) and a prospective observational evaluation (SAQ2).

SAQ1 assessed baseline demographic and treatment information, hypoglycemia unawareness and perceptions of hypoglycemia, history of severe hypoglycemia for 6 months before the baseline visit, and “any” and “nocturnal” hypoglycemia for 4 weeks before the baseline visit.

SAQ2 assessed severe and symptomatic hypoglycemia and its effect on productivity and healthcare utilization for 4 weeks from the baseline visit.

SAQ2 also included a validated diabetes-specific quality of life (DSQOL) questionnaire. To assist patients recall, and as a reminder to complete SAQ2, patients were provided with a diary to capture hypoglycemic episodes. Paired responses to SAQ1 and SAQ2 were used to estimate the differences in the frequency of hypoglycemic episodes between the retrospective and prospective periods. The incidence of severe and symptomatic hypoglycemia (defined below) was calculated according to the frequency of episodes over the timeframe stated in the corresponding question. The diary which allowed patients to summarize hypoglycemia information on a daily basis over the 4-week period following the baseline visit was used to evaluate the incidence of hypoglycemia. If there were discrepancies between the diary and the SAQ2 questionnaire, the frequency of hypoglycemia was calculated using the highest recorded total frequency as stated on either of these forms.

Hypoglycemia unawareness was evaluated through the question: ‘Do you have symptoms when you have a low sugar level?’ where the response, ‘occasionally’ denoted impaired awareness and ‘never’ denoted severely impaired awareness [12]. Fear of hypoglycemia was reported as rated by the patient on a scale of 0 (not afraid at all) to 10 (absolutely terrified).

### Study objectives

The primary objective of the study was to determine the percentage of patients experiencing at least 1 hypoglycemic episode during the 4-week prospective observational period among insulin-treated T1DM or T2DM patients.

The secondary objectives included: incidence of hypoglycemic episodes, difference in the incidence of hypoglycemic episodes before and after the baseline visit, relationship between patient demography, treatment, and hypoglycemia, use of health-care resources, and types of behaviors against hypoglycemia. Diabetes-related late complications, treatment regimen and glycemic control were ascertained from questions completed in the presence of the participant’s health-care professional to improve accuracy.

All other study end-points including the primary endpoint of interest were determined from questions completed by the patient.

### Hypoglycemia classification

Severe hypoglycemia was defined as: requiring third-party assistance, based on the American Diabetes Association (ADA) definition [13]; non-severe hypoglycemia: managed by patient alone; any hypoglycemia: the sum of severe and non-severe hypoglycemia; nocturnal hypoglycemia: event occurring between midnight and 06:00 h.

### Sample size

Sample size of the total cohort across 9 countries of the IO HAT study was determined to be 6000 patients assuming a worst case scenario proportion of patients (50%) reporting at least 1 hypoglycemic episode during the 4-week prospective observation period, and that the range of the 95% confidence interval was < 3 percentage point for the total cohort. Of these, 2000 patients were planned to be recruited from Turkey.

### Evaluability of patients for analysis

Patients who returned any part of any SAQ or patient diary containing answers to any of the questions received was also included in the Full Analysis Set (FAS).

### Statistical methods

All statistical tests were two-sided and regarded as exploratory, with the criterion for statistical significance set at *p* < 0.05. The *p*-values from 0.01 to 0.05 were taken to indicate a modest evidence of a difference, and p-values of < 0.01 were taken to indicate moderate evidence.

For the primary endpoint, the percentage of patients who experienced at least 1 hypoglycemic episode during the 4-week prospective observational period among T1DM or T2DM patients was calculated together with the confidence interval for this percentage. For secondary endpoints, the incidence of various types of hypoglycemia was calculated as number of episodes per patient-year (ppy) as expressed by the following formula (together with the 95% confidence interval).

Incidence rate = Total number of events / Total follow-up time (patient-years).

The incidence rate (IR) was reported by diabetes type: T1DM and T2DM patients. No imputation of missing data was performed except for calculation of Well-Being Questionnaire-5 summary scores where more than half the items were non-missing. All analyses were conducted in the FAS.

Relationship between HbA_1c_ at baseline and log-transformed number of hypoglycemia events reported by patients was shown by the scatter plot with regression line and 95% confidence interval (CI) and R-squared values were calculated.

Baseline refers to data collected using the Part 1 SAQ; follow-up refers to data collected using the Part 2 SAQ and, where applicable, patient diaries.

## Results

### Patient characteristics

Overall, 2348 patients (306 with T1DM and 2042 with T2DM) from Turkey enrolled and completed the Part 1 SAQ in the Turkey cohort constituting the FAS. Of these, 252 patients (82.4%) with T1DM and 1781 patients (87.2%) with T2DM completed the Part 2 SAQ; and 247 patients (80.7%) with T1DM and 1749 patients (85.7%) with T2DM completed the patient diary and were included in the completers analysis set (CAS).

Baseline characteristics for T1DM and T2DM patients in the FAS are presented in Table 1. Patients with T1DM were younger than those with T2DM (32.7 years vs. 58.0 years, respectively) and had a longer median duration of insulin use (11.2 years vs. 6.0 years, respectively). Mean HbA_1c_ was lower in patients with T1DM (8.4% [67.9 mmol/mol]) than in those with T2DM (8.8% [72.3 mmol/mol]).

**Table 1:** Baseline characteristics

	T1DM (*N* = 306)	T2DM (*N* = 2042)
Age (years)	32.7 (11.6)	58.0 (10.5)
Median	30.5	58.0
Upper quartile, Lower quartile	39.0, 24.0	65.0, 51.0
Male/female (%)	44.1/55.9	40.9/59.1
Duration of diabetes (years)	12.1 (8.0)	12.5 (7.0)
Median	10.5	11.0
Upper quartile, lower quartile	17.0, 6.0	17.0, 7.0
Duration of insulin use (years)	11.2 (7.8)	6.0 (4.6)
Median	10.0	5.0
Upper quartile, lower quartile	16.0, 5.0	8.0, 3.0
HbA_1c_ (mmol/mol)	67.9 (18.1)	72.3 (20.4)
HbA_1c_ (%)	8.4 (1.7)	8.8 (1.9)
FBG (mmol/L)	8.6 (4.1)	9.6 (4.0)
FBG (mg/dL)	155.0	173.0
PPG (mmol/L)	11.1 (4.8)	12.5 (4.9)
PPG (mg/dL)	200.0 (86.5)	225.2 (88.3)
Weight (kg)	70.4 (16.2)	83.7 (14.9)
Median	68.0	82.0
Upper quartile, lower quartile	80.0, 60.0	92.0, 74.0
Height (cm)	167.8 (10.0)	164.2 (8.5)
Median	168.0	164.0
Upper quartile, lower quartile	174.0, 160.0	170.0, 158.0
BMI (kg/m^2^)	25.0 (5.2)	31.2 (5.7)
Median	24.3	30.5
Upper quartile, lower quartile	27.2, 21.8	34.0, 27.3
Previous medical illnesses	% of patients	% of patients
Neuropathy	34.6	53.1
Retinopathy	20.6	39.6
Nephropathy	9.2	14.6
Peripheral vascular disease	14.7	20.2
Angina	9.8	17.4
Myocardial infarction	3.3	15.6
None	49.0	27.7
Symptoms of diabetes-related complications, %		
Any	98.4	95.4
Tremor	84.0	80.5
Sweating	85.6	79.4
Hunger	83.0	78.2
Tiredness	83.0	76.4
Weakness	78.4	72.9
Diabetes treatment regimen, %		
Short-acting insulin	12.1	5.5
Long-acting insulin	4.6	17.3
Pre-mix	4.2	33.3
Both short- and long-acting	74.2	41.1
Both short-acting and pre-mix	0.3	0.6
Both long-acting and pre-mix	3.3	1.4
Short- and long-acting and pre-mix	0.0	0.0
Missing	1.3	0.8

Data are presented as mean (SD) unless otherwise stated

BMI = body mass index, FBG = fasting blood glucose, HbA1c = glycated hemoglobin, N = total number of patients participating, PPG = postprandial glucose, SD = standard deviation, T1DM = type I diabetes mellitus, T2DM = type II diabetes mellitus

### Frequency of hypoglycemia

#### Any hypoglycemia

**Fig. 2:**
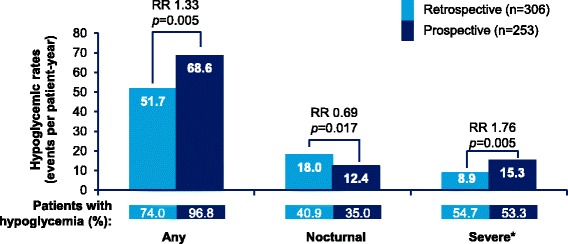
Estimated rate of retrospective and prospective hypoglycemia in T1DM (any, nocturnal, and severe hypoglycemia). ‘Any’ and ‘Nocturnal’ based on 4-week period for both retrospective and prospective analyses. *Retrospective data based on 6-month period and prospective data based on 4-week period. RR = rate ratio; T1DM = type I diabetes mellitus

**Fig. 3:**
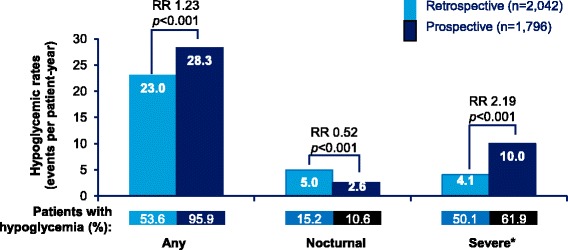
Estimated rate of retrospective and prospective hypoglycemia in T2DM (any, nocturnal, and severe hypoglycemia). ‘Any’ and ‘Nocturnal’ based on 4-week period for both retrospective and prospective analyses. *Retrospective data based on 6-month period and prospective data based on 4-week period. RR = rate ratio; T2DM = type II diabetes mellitus

Any hypoglycemia rates in T1DM and T2DM patients are presented in Figs. 2 and 3, respectively. In T1DM patients, 96.8% patients reported hypoglycemic events (IR: 68.6 events ppy), prospectively, while 74.0% patients reported hypoglycemic events (IR: 51.7 events ppy), retrospectively. In T2DM patients, 95.9% patients (IR: 28.3 events ppy) reported hypoglycemic events, prospectively, while 53.6% patients (IR: 23.0 events ppy) reported hypoglycemic events, retrospectively.

The rates of any hypoglycemia were significantly higher in the prospective period compared with the retrospective period in both T1DM and T2DM patients (T1DM, *p* = 0.005; T2DM, *p* < 0.001).

#### Nocturnal hypoglycemia

Nocturnal hypoglycemia rates in T1DM and T2DM patients are presented in Figs. 2 and 3, respectively. Unlike, any hypoglycemia, the retrospective rates for nocturnal hypoglycemia were higher compared to the prospective rates in both T1DM and T2DM patients; 40.9% vs. 35.0% (IR: 18.0 ppy vs. 12.4 ppy) in T1DM, 15.2% vs. 10.6% (IR:5.0 ppy vs. 2.6 ppy) in T2DM; (T1DM, *p* = 0.017; T2DM, *p* < 0.001).

#### Severe hypoglycemia

Severe hypoglycemia rates in T1DM and T2DM patients are presented in Figs. 2 and 3, respectively. In T1DM patients, in the 6-month retrospective period, severe hypoglycemia was reported by 54.7% (IR: 8.9 events ppy) patients, while in the 4-week prospective period, severe hypoglycemia was reported by 53.3% (IR: 15.3 events ppy) patients. In T2DM patients, 50.1% (IR: 4.1 events ppy) and 61.9% (IR: 10.0 events ppy) patients reported severe hypoglycemia, retrospectively and prospectively. The rates of severe hypoglycemia were significantly higher in the prospective period compared with those in the retrospective period (T1DM, *p* = 0.005; T2DM, p < 0.001).

### Use of health system resources

The impact of hypoglycemia on the medical system was higher in the 6-month retrospective period than in the 4-week prospective period for both T1DM and T2DM patients (Table 2).

**Table 2:** Patient perspectives on hypoglycemia

	T1DM		T2DM	
Impact of hypoglycemic events on the medical system (%)	Retrospective (n = 306)	Prospective (n = 253)	Retrospective (n = 2042)	Prospective (n = 1796)
Events requiring hospital admission	10.2	3.3	6.1	1.9
Attended additional clinical appointments	15.8	12.6	11.4	8.0
Made additional telephone contacts	6.3	5.3	4.1	3.3
Patient response to hypoglycemia (%)	Retrospective (n = 306)	Prospective (n = 252)	Retrospective (n = 2042)	Prospective (n = 1781)
Consulted their doctor/nurse	47.4	32.9	39.3	32.6
Required any form of medical assistance	48.7	33.3	41.0	32.6
Increased calorie intake	35.3	28.6	27.6	17.6
Avoided physical exercise	17.6	13.1	11.3	9.2
Reduced insulin dose	35.0	18.7	18.8	13.0
Skipped insulin injections	23.9	10.3	18.0	9.8
Increased blood glucose monitoring	52.3	46.4	28.9	20.9
Impact of hypoglycemic events on work and study (%)	Retrospective (n = 200)	Prospective (n = 166)	Retrospective (n = 360)	Prospective (n = 321)
Taken leave from work or studies	22.0	6.6	10.8	3.1
Arrived late to work/studies	24.5	9	6.4	2.5
Left early from work/studies	20.5	11.4	10.8	2.5
	T1DM (N = 306)		T2DM (N = 2042)	
Knew what hypoglycemia was at baseline before Part 1 SAQ (%)				
	91.3		60.4	
Defined hypoglycemia based on (%)				
Symptoms only	54.6		52.4	
Blood glucose measurement only	2.3		3.5	
Either	15.4		15.2	
Both	24.2		18.8	
Hypoglycemia awareness (%)				
Normal	71.6		53.3	
Impaired	25.2		38.5	
Severely impaired	0.7		2.8	
Fear of hypoglycemia (Scale of 0 to 10; %)				
0 = no fear	19.9		24.3	
1	3.9		5.9	
2	2.3		5.8	
3	5.6		5.4	
4	5.6		4.6	
5	13.1		10.7	
6	6.5		6.6	
7	9.8		9.9	
8	10.1		8.7	
9	5.2		4.6	
10 = absolutely terrified	17.3		12.2	

Hypoglycemia unawareness was evaluated through the question: ‘Do you have symptoms when you have a low sugar level?’ where the response, ‘occasionally’ denoted impaired awareness and ‘never’ denoted severely impaired awareness

N = total number of patients participating; n = number of patients who responded to the set of questions; SAQ = self-assessment questionnaire; T1DM = type I diabetes mellitus; T2DM = type II diabetes mellitus

#### Hypoglycemia requiring hospitalization

In T1DM patients, 10.2% patients (6-month retrospective period) and 3.3% patients (4-week prospective period) reported hypoglycemia requiring hospitalization. In T2DM patients, 6.1% patients (6-month retrospective period) and 1.9% patients (4-week prospective period) reported hypoglycemia requiring hospitalization (Table 2).

#### Requiring additional clinic appointments

In T1DM patients, 15.8% (6-month retrospective period) and 12.6% (4-week prospective period) patients required additional clinic appointments. In T2DM patients, 11.4% (6-month retrospective period) and 8.0% (4-week prospective period) required additional clinic appointments (Table 2).

#### Requiring number of additional telephone contacts made

In T1DM patients, 6.3% patients (6-month retrospective period) and 5.3% patients (4-week prospective period) made additional telephone contacts. In T2DM patients, 4.1% patients (6-month retrospective period) and 3.3% patients (4-week prospective period) made additional telephone contacts (Table 2).

### Patient response to hypoglycemia

The overall patient actions resulting from hypoglycemia were more in the 6-month retrospective period than in the 4-week prospective period in both T1DM and T2DM patients (Table 2). For patients with T1DM, the 6-month retrospective and 4-week prospective data, respectively, were: the percentage of patients who consulted their doctor or nurse (47.4% vs. 32.9%), required any form of medical assistance (48.7% vs. 33.3%), increased calorie intake (35.3% vs. 28.6%), avoided physical exercise (17.6% vs. 13.1%), reduced insulin dose (35.0% vs. 18.7%), skipped insulin injections (23.9% vs. 10.3%), and increased blood glucose monitoring (52.3% vs. 46.4%).

In the T2DM patients, the 6-month retrospective and 4-week prospective data, respectively, were: the percentage of patients who consulted their doctor or nurse (39.3% vs. 32.6%), required any form of medical assistance (41.0% vs. 32.6%), increased calorie intake (27.6% vs. 17.6%), avoided physical exercise (11.3% vs. 9.2%), reduced insulin dose (18.8% vs. 13.0%), skipped insulin injections (18.0% vs. 9.8%), and increased blood glucose monitoring (28.9% vs. 20.9%).

### Impact of hypoglycemia on work/studies

Higher percentage of patients took leave from work/studies, arrived late or left early from work/studies in the retrospective period than the prospective period (Table 2). In T1DM patients, the 6-month retrospective and 4-week prospective data, respectively were: 22.0% vs. 6.6% patients had taken leave from work/studies, 24.5% vs. 9.0% patients had arrived late to work/studies, and 20.5% vs. 11.4% patients left early from work/studies. In T2DM patients, the 6-month retrospective and 4-week prospective data, respectively were: 10.8% vs. 3.1% patients had taken leave from work/studies, 6.4% vs. 2.5% patients had arrived late to work/studies, and 10.8% vs. 2.5% patients left early from work/studies.

### Hypoglycemia awareness

More patients with T1DM than with T2DM had knowledge of hypoglycemia before reading the definition in the Part 1 SAQ (91.3% [T1DM] and 60.4% [T2DM]) and had a higher normal hypoglycemia awareness (71.6% [T1DM] and 53.3% [T2DM]) (Table 2). There were no notable differences between patients with T1DM or T2DM with respect to fear of hypoglycemia, with a mean (standard deviation) score of 5.3 (3.6) for patients with T1DM and 4.5 (3.6) for patients with T2DM (Table 2).

### Hypoglycemia by insulin regimen

**Fig. 4:**
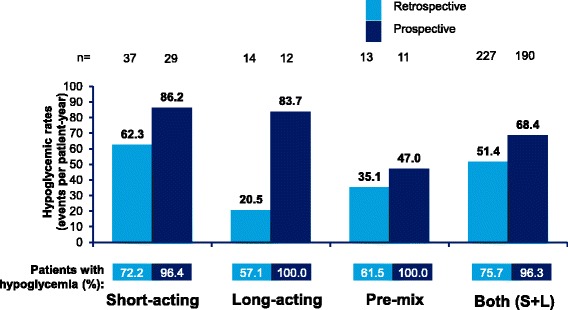
Estimated rate of any hypoglycemic event by insulin regimen in T1DM. Data based on 4-week period for both retrospective and prospective analyses. S + L = short-acting and long-acting insulin; T1DM = type I diabetes mellitus; n = number of patients

**Fig. 5:**
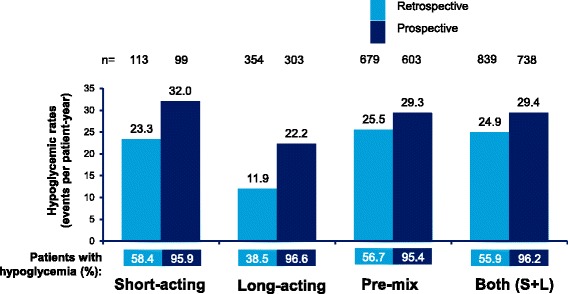
Estimated rate of any hypoglycemic event by insulin regimen in T2DM. Data based on 4-week period for both retrospective and prospective analyses. S + L = short-acting and long-acting insulin; T2DM = type 2 diabetes mellitus; n = number of patients

Incidence rates of any hypoglycemia in T1DM and T2DM patients in the 4-week retrospective and prospective assessment periods by insulin regimen (short-acting, long-acting, pre-mix, and short- plus long-acting) are shown in Figs. 4 and 5, respectively. Estimated IRs of any and severe hypoglycemia increased whilst estimated IRs of nocturnal hypoglycemia generally decreased in the prospective period versus the retrospective period in patients with T1DM and T2DM.

The estimated IRs of any hypoglycemic events in the 4-week retrospective and 4-week prospective assessment were highest in patients with T1DM using short-acting insulin in the prospective period (86.2 ppy) and lowest in patients with T2DM using long-acting insulin, in the retrospective period (11.9 ppy).

The IRs of nocturnal hypoglycemia were highest in patients with T1DM using short-acting insulin in the 4-week retrospective period (20.5 events ppy) and lowest in the 4-week prospective period in T2DM patients using short-acting insulin (1.1 events ppy).

The IRs of severe hypoglycemia were highest in T1DM patients using pre-mix insulin in the 4-week prospective period (19.6 ppy) and lowest in T1DM patients using long-acting insulin in the 6-month retrospective period (1.2 ppy).

### Associations between hypoglycemia and continuous or predictor variables

**Fig. 6:**
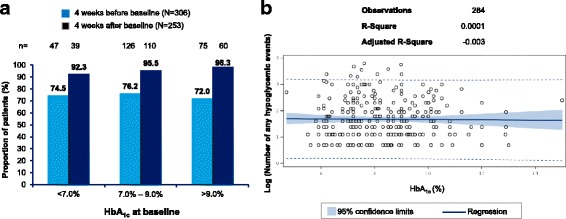
Relationship between HbA_1c_ and number of events – any hypoglycemic event in T1DM. **a** Proportion of patients experiencing any hypoglycemia during the retrospective and prospective periods, stratified by HbA_1c_ levels at baseline **b** Scatter plot with regression line and 95% confidence interval for relationship between HbA_1c_ at baseline and log-transformed number of events for patients experiencing any hypoglycemia before or after baseline. HbA_1c_ = hemoglobin A_1c_; T1DM = type I diabetes mellitus

**Fig. 7:**
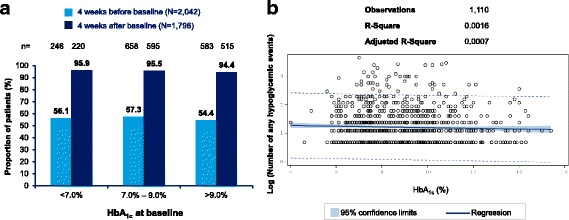
Relationship between HbA_1c_ and number of events – any hypoglycemic event in T2DM. **a** Proportion of patients experiencing any hypoglycemia during the retrospective and prospective periods, stratified by HbA_1c_ levels at baseline **b** Scatter plot with regression line and 95% confidence interval for relationship between HbA_1c_ at baseline and log-transformed number of events for patients experiencing any hypoglycemia before or after baseline. HbA_1c_ = hemoglobin A_1c_; T2DM = type I diabetes mellitus

**Fig. 8:**
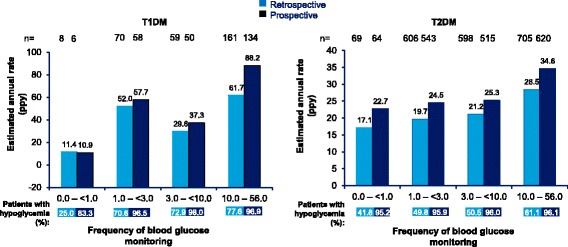
Estimated rate of any hypoglycemic event by glucose monitoring frequency in T1DM and T2DM patients. Percentages represent percent of patients with hypoglycemia in each quartile. PPY = per patient-year; T1DM = type 1 diabetes mellitus; T2DM = type 2 diabetes mellitus

In this study, no correlation was observed between baseline HbA_1c_ and any hypoglycemia events in both T1DM and T2DM populations (Figs. 6 and 7, respectively). No significant association between hypoglycemia and duration of diabetes or duration of insulin therapy was seen (Additional files 1 and 2, respectively). Patients who measured their blood glucose levels more frequently reported higher rates of hypoglycemia compared to those who monitored their blood glucose levels less frequently (Fig. 8).

## Discussion

This paper describes the results from the Turkey cohort of the international, non-interventional, multicenter, retrospective and prospective study to assess the incidence of patient-reported hypoglycemia in insulin-treated diabetes patients.

This is a first report of an observational study to assess hypoglycemia both retrospectively and prospectively in the Turkish T1DM and T2DM patients. While hypoglycemia has been reported from a few observational studies in Turkish population, the main aim of these studies were not to assess hypoglycemia. Minor hypoglycemia rates of 1.08 and 2.56 ppy were prospectively observed in the insulin detemir and insulin glargine group, respectively, in the Turkish T2DM cohort from the observational SOLVE study [10]. The lower hypoglycemic frequencies obtained in these studies could be explained by the fact that hypoglycemia assessment was not the primary objective of these studies and the reported frequency is for minor hypoglycemia which may not encompass a total hypoglycemic rate. Total hypoglycemic episodes in the 4-week retrospective period in the PREDICTIVE study were 47.5 ppy in patients with T1DM and 9.2 ppy in patients with T2DM in the European cohort (Turkey population was included) [14], which are comparable to those for T1DM population but less compared to T2DM population observed in the current study (51.7 ppy and 23.0 ppy, respectively).

The hypoglycemia rates seen in the IO HAT Turkey cohort aligned with the overall IO HAT results [15]. The frequency of overall hypoglycemia in the prospective period in the Turkey cohort was comparable to global HAT in T1DM patients but was considerably higher in T2DM patients (28.3 events ppy) than the global HAT study (19.3 events ppy). The reason for this could be the country-specific variations in the prevalence and management of diabetes and hypoglycemia in the two studies.

Similar to the overall IO HAT results, higher frequency of patients reported hypoglycemia in the prospective period as compared to the retrospective period in both T1DM and T2DM patients in the Turkey cohort. The reason for this could be the use of patient diary during the prospective period. While a patient recorded data daily using patient diary during the prospective period, the data for the retrospective period was collected at baseline visit based on the patient’s memory of the previous hypoglycemic events, possibly causing under-reporting. The patient education on hypoglycemia at the baseline visit could have also led to an improved reporting of hypoglycemia in the prospective period. On similar lines, the higher frequency of severe hypoglycemia observed during the prospective period over the retrospective period in both T1DM and T2DM patients could also be explained. However, a lower frequency of nocturnal hypoglycemia was reported in the prospective period over the retrospective period. This could be because of a well-defined cut-off for the nocturnal hypoglycemia, midnight to 06.00 am, during the prospective period. The perceived fear of nocturnal hypoglycemia could also probably cause an over-reporting of events during the retrospective period based on patient recall. Also, difficulty in using a diary during the night-time could have affected the reporting of nocturnal hypoglycemia during the prospective period. Interestingly, though the prospectively reported “any” and “severe” hypoglycemia rates were higher than retrospectively reported rates, a higher proportion of patients reported increased utilization of healthcare resources (hospital admissions, additional clinical appointments) in the retrospective period than the prospective period. Similarly, a higher proportion of patients reported that the hypoglycemic events impacted their work and study in the retrospective period than in the prospective period. The reason for this could be because the patients were well-informed about hypoglycemia at the baseline visit leading to less impact on patients’ quality of life in the prospective period. Another explanation could be that the assessment period for some of the parameters in the retrospective period was of 6 months compared to 4 weeks during the prospective period and hence the difference.

In the PREDICTIVE study, the frequency of hypoglycemia in insulin-treated patients showed a significant, positive association with duration of diabetes, and number of insulin injections but was inversely related to HbA_1c_ [14]. Unlike the global HAT study [11], no significant correlation of hypoglycemia with duration of diabetes and insulin therapy was seen in the current study. Also, no significant correlation between HbA_1c_ and hypoglycemia was observed which is in line with global HAT study results [11] and recent findings that the inverse correlation between HbA_1c_ and hypoglycemia has diminished due to advances in therapy in the recent years [16]. A regular self-monitoring of blood glucose is important to detect hypoglycemia and for overall diabetes management [17]. A positive correlation between frequency of blood glucose monitoring and reported hypoglycemia rates was seen in the current study which suggests its importance to detect hypoglycemia.

## Conclusions

The current study has enabled to obtain real-world data on hypoglycemia rates from Turkey where very few data were available in spite of a high rate of diabetes prevalence. The results from this study confirms that hypoglycemia remains under-reported. The higher hypoglycemic rates observed in Turkish population could be because of higher burden of diabetes combined with lack of standard care and treatment as compared to European and North American population and needs to be investigated further. The hypoglycemia data in Turkish cohort is an important step towards a customized country-specific healthcare plan to control diabetes.

## Additional file

Additional file 1: Estimated rate of any hypoglycemic event by duration of diabetes in T1DM and T2DM patients. Percentages represent percent of patients with hypoglycemia in each quartile. PPY = per patient-year; T1DM = type 1 diabetes mellitus; T2DM = type 2 diabetes mellitus. (PPTX 361 kb)

Additional file 2: Estimated rate of any hypoglycemic event by duration of insulin therapy in T1DM and T2DM patients. Percentages represent percent of patients with hypoglycemia in each quartile. PPY = per patient-year; T1DM = type 1 diabetes mellitus; T2DM = type 2 diabetes mellitus. (PPTX 362 kb)

## Abbreviations

ADA: American Diabetes Association
CAS: Completers analysis set
CI: Confidence interval
DSQOL: Diabetes-related quality of life
FAS: Full analysis set
IO HAT: International Operations Hypoglycemia Assessment Tool
IR: Incidence rate
ppy: Per patient-year
QOL: Quality of life
SAQ: Self-assessment questionnaire
T1DM: Type I diabetes mellitus
T2DM: Type II diabetes mellitus
