# Supplementary material for: An international survey on hypoglycemia among insulin-treated type I and type II diabetes patients: Turkey cohort of the non-interventional IO HAT study
Source: BMC Endocr Disord. 2018 Feb 13;18:9. doi: 10.1186/s12902-018-0238-2 (PMC5809967; doi:10.1186/s12902-018-0238-2)
Supplement: Supplementary file 1 — Estimated rate of any hypoglycemic event by duration of diabetes in T1DM and T2DM patients. Percentages represent percent of patients with hypoglycemia in each quartile. PPY = per patient-year; T1DM = type 1 diabetes mellitus; T2DM = type 2 diabetes mellitus. (PPTX 361 kb) [file 12902_2018_238_MOESM1_ESM.pptx]

## Slide 1
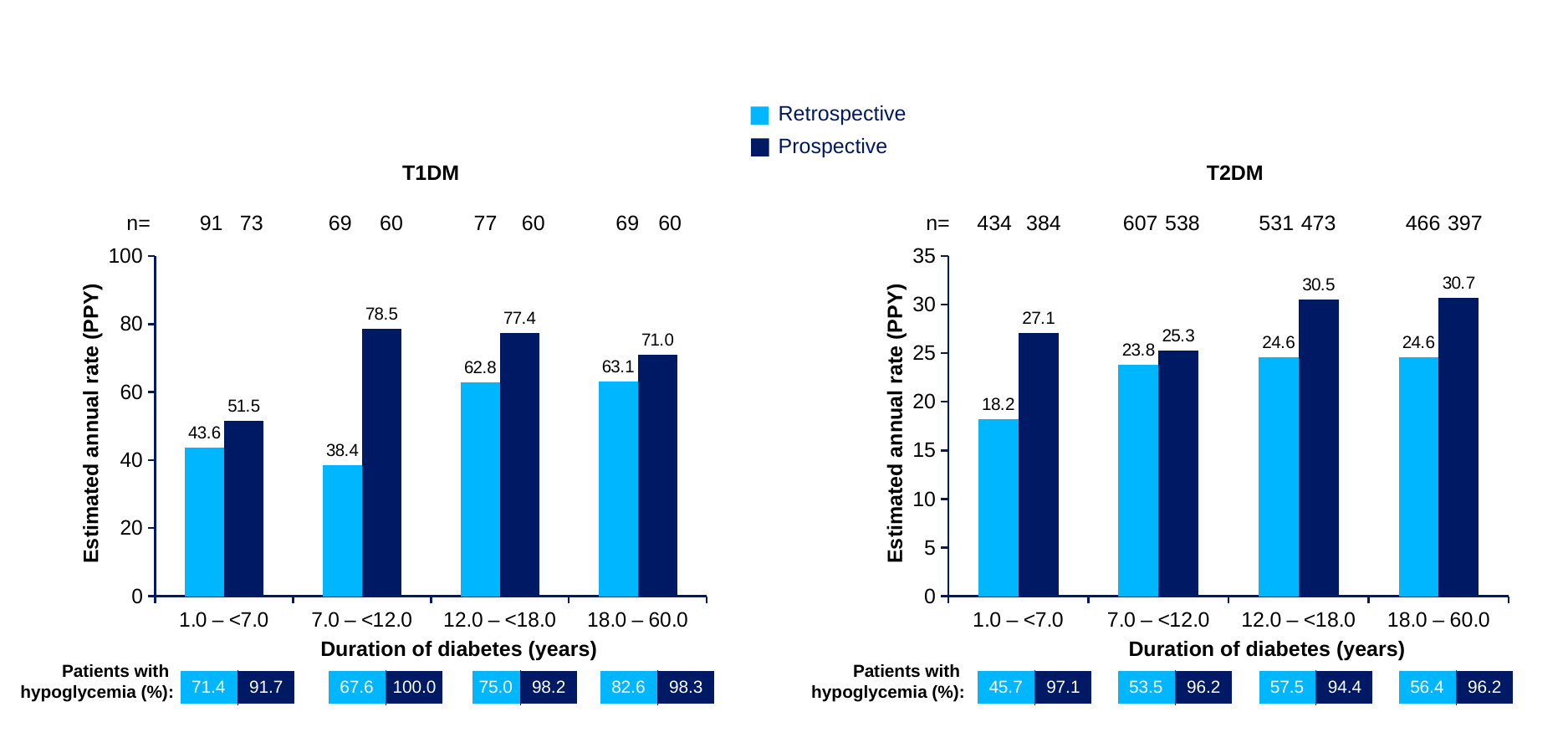

Retrospective
Prospective
 T1DM
 T2DM
### Chart
| Category | Retrospecitve | Prospective |
|---|---|---|
| 1.0 – <7.0 | 43.6 | 51.5 |
| 7.0 – <12.0 | 38.4 | 78.5 |
| 12.0 – <18.0 | 62.8 | 77.4 |
| 18.0 – 60.0 | 63.1 | 71.0 |
### Chart
| Category | Retrospective | Prospective |
|---|---|---|
| 1.0 – <7.0 | 18.2 | 27.1 |
| 7.0 – <12.0 | 23.8 | 25.3 |
| 12.0 – <18.0 | 24.6 | 30.5 |
| 18.0 – 60.0 | 24.6 | 30.67 |n=
434
384
607
538
531
473
466
397
n=
91
73
69
60
77
60
69
60
Estimated annual rate (PPY)
Estimated annual rate (PPY)
Duration of diabetes (years)
Duration of diabetes (years)
Patients with
hypoglycemia (%):
Patients with
hypoglycemia (%):
| 82.6 | 98.3 |
| --- | --- |
| 45.7 | 97.1 |
| --- | --- |
| 53.5 | 96.2 |
| --- | --- |
| 57.5 | 94.4 |
| --- | --- |
| 56.4 | 96.2 |
| --- | --- |
| 71.4 | 91.7 |
| --- | --- |
| 67.6 | 100.0 |
| --- | --- |
| 75.0 | 98.2 |
| --- | --- |
