# Supplementary material for: An international survey on hypoglycemia among insulin-treated type I and type II diabetes patients: Turkey cohort of the non-interventional IO HAT study
Source: BMC Endocr Disord. 2018 Feb 13;18:9. doi: 10.1186/s12902-018-0238-2 (PMC5809967; doi:10.1186/s12902-018-0238-2)
Supplement: Supplementary file 2 — Estimated rate of any hypoglycemic event by duration of insulin therapy in T1DM and T2DM patients. Percentages represent percent of patients with hypoglycemia in each quartile. PPY = per patient-year; T1DM = type 1 diabetes mellitus; T2DM = type 2 diabetes mellitus. (PPTX 362 kb) [file 12902_2018_238_MOESM2_ESM.pptx]

## Slide 1
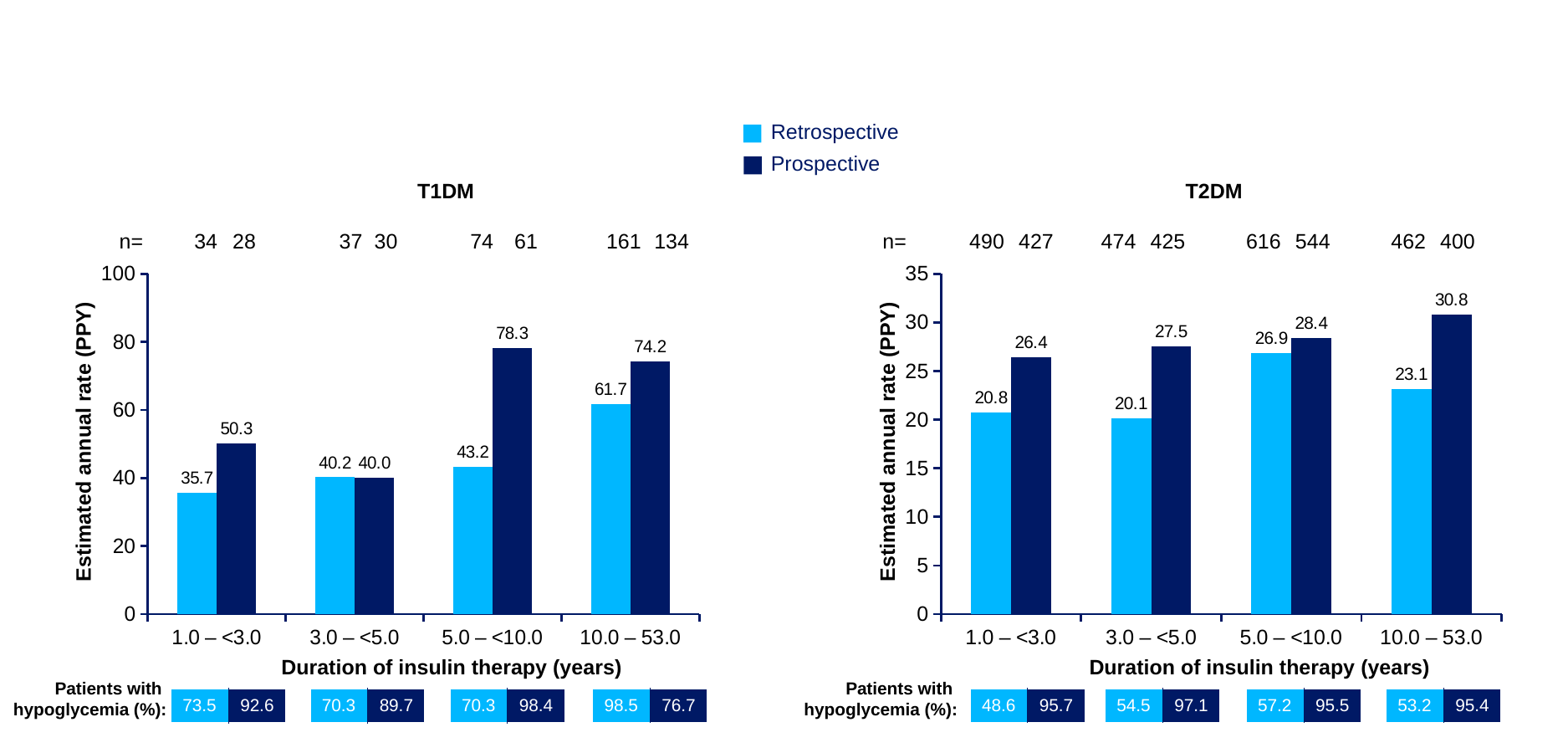

Retrospective
Prospective
 T1DM
T2DM
### Chart
| Category | Retrospecitve | Prospective |
|---|---|---|
| 1.0 – <3.0 | 35.68 | 50.25 |
| 3.0 – <5.0 | 40.190000000000005 | 40.03 |
| 5.0 – <10.0 | 43.190000000000005 | 78.27 |
| 10.0 – 53.0 | 61.7 | 74.19 |
### Chart
| Category | Retrospective | Prospective |
|---|---|---|
| 1.0 – <3.0 | 20.779999999999998 | 26.43 |
| 3.0 – <5.0 | 20.09 | 27.52 |
| 5.0 – <10.0 | 26.88 | 28.39 |
| 10.0 – 53.0 | 23.14 | 30.8 |n=
490
427
474
425
616
544
462
400
n=
34
28
37
30
74
61
161
134
Estimated annual rate (PPY)
Estimated annual rate (PPY)
Duration of insulin therapy (years)
Duration of insulin therapy (years)
Patients with
hypoglycemia (%):
Patients with
hypoglycemia (%):
| 98.5 | 76.7 |
| --- | --- |
| 48.6 | 95.7 |
| --- | --- |
| 54.5 | 97.1 |
| --- | --- |
| 57.2 | 95.5 |
| --- | --- |
| 53.2 | 95.4 |
| --- | --- |
| 73.5 | 92.6 |
| --- | --- |
| 70.3 | 89.7 |
| --- | --- |
| 70.3 | 98.4 |
| --- | --- |
